# MolData, a molecular benchmark for disease and target based machine learning

**DOI:** 10.1186/s13321-022-00590-y

**Published:** 2022-03-07

**Authors:** Arash Keshavarzi Arshadi, Milad Salem, Arash Firouzbakht, Jiann Shiun Yuan

**Affiliations:** 1 Burnett School of Biomedical Sciences, University of Central Florida, Orlando, FL USA; 2 Department of Electrical and Computer Engineering, University of Central Florida, Orlando, FL USA; 3 Department of Chemistry, University of Illinois at Urbana, Champaign, IL USA

**Keywords:** Artificial intelligence, Benchmark, Biological assays, Big data, Database, Drug discovery, Machine learning, PubChem

## Abstract

Deep learning’s automatic feature extraction has been a revolutionary addition to computational drug discovery, infusing both the capabilities of learning abstract features and discovering complex molecular patterns via learning from molecular data. Since biological and chemical knowledge are necessary for overcoming the challenges of data curation, balancing, training, and evaluation, it is important for databases to contain information regarding the exact target and disease of each bioassay. The existing depositories such as PubChem or ChEMBL offer the screening data for millions of molecules against a variety of cells and targets, however, their bioassays contain complex biological descriptions which can hinder their usage by the machine learning community. In this work, a comprehensive disease and target-based dataset is collected from PubChem in order to facilitate and accelerate molecular machine learning for better drug discovery. MolData is one the largest efforts to date for democratizing the molecular machine learning, with roughly 170 million drug screening results from 1.4 million unique molecules assigned to specific diseases and targets. It also provides 30 unique categories of targets and diseases. Correlation analysis of the MolData bioassays unveils valuable information for drug repurposing for multiple diseases including cancer, metabolic disorders, and infectious diseases. Finally, we provide a benchmark of more than 30 models trained on each category using multitask learning. MolData aims to pave the way for computational drug discovery and accelerate the advancement of molecular artificial intelligence in a practical manner. The MolData benchmark data is available at https://GitHub.com/Transilico/MolData as well as within the additional files.

**Supplementary Information:**

The online version contains supplementary material available at 10.1186/s13321-022-00590-y.

## Introduction

In the last decade, Artificial Intelligence (AI) has played a major role in modern computer aided drug discovery (CADD). Major improvements in both structure-based and ligand-based virtual screening have been recorded by training smart systems capable of identifying hidden molecular patterns [1–3]. Training models for Ligand-Based Drug Discovery (LBDD), or non-structural drug discovery, have been truly revolutionary in multiple aspects of the early drug discovery process. Deep Learning (DL) models have demonstrated the ability to discover abstract features of small molecules, allowing for better screening of both cell-based and target-based CADD. Using conventional methods, scientists would need to screen every molecule in a library on a specific target or cell, which is expensive, labor intensive, and time consuming. Virtual screening algorithms have introduced more affordable and faster alternatives that eliminate most of the early drug discovery costs. However, despite advances in CADD, the accuracy of traditional molecular modeling methods in most cases had not been satisfactory, prior to the introduction of Machine Learning (ML). Automatic feature extraction from molecules and learning of hidden patterns in a large molecular library, are just some examples of what AI has changed forever in the drug discovery field [1, 4].

One of the most important factors of a reliable model is its training data, and deep learning models utilize this data to automate both pattern extraction and the prediction of bioactive molecules [5, 6]. In general, datasets that are large, more diverse, and less biased result in training smarter systems with better inner features, performance, and generalization. Therefore, the first goal for machine learning scientists should be identifying and curating the right dataset per disease state. This curation can include selection of bioassays that relate to the biological question at hand typically guided by the description of the bioassays, cleaning the molecular data and dealing with the sparse and missing values within the molecular dataset. In addition, understanding the biological knowledge behind a dataset is as important as the data quality. Since data curation, model training, and model evaluation are time consuming and tedious, it is crucial to know the exact applications of the biological target for the disease of interest. To this end, current input datasets need to be improved upon. Firstly, biomedical datasets tend to be very biased and imbalanced based on the biological assay and the chemical library [7]. Secondly, understanding the exact cellular and molecular background and description of the assays requires expert knowledge that ML scientists or cheminformaticians might not possess. Without knowing the biological background of the data, it would be difficult to devise solutions for data balancing and model evaluation. This knowledge is also necessary for finding appropriate public datasets due to their complicated descriptions and goals. Lastly, the chemical diversity, druggability, and toxicity of the predicted molecules need to be investigated [8, 9]. With the emergence of AI in non-structural drug discovery, there has been a renewed need for cleaned and clustered public molecular databases with simple and sufficient biological information, including the proper disease and targets involved in each bioassay.

There are multiple molecular depositories containing millions of molecules and hundreds of thousands of bioassays for specific biomedical aims. PubChem bioassays [10], ChEMBL datasets [12], and ChemSpider [13] are among some of the most comprehensive and well-known examples. These databases collect large sets of molecular activity outcomes for specific cells or protein targets. Even though these databases are excellent resources for model training, discovering the right bioassays and categorizing them based on disease, targets, and signaling pathways can often be challenging and non-intuitive due to their non-standardized descriptions [14, 15]. However, the scientific community persists and has been benchmarking datasets and methods with these depositories, and in-house databases, in order to facilitate their usage and accelerate the advancement of molecular machine learning [16]. To do so, researchers often curate, analyze, and publish datasets with intended targets for discovering specific patterns in bioactive molecules. One of the first examples would be the ‘Merck Molecular Activity Challenge’ which had 15 biological assay tasks. In this dataset, targets are selected based on their molecular classes [17]. In toxicity field, the Tox21 dataset from National Center for Advancing Translational Science (NCATS) containing 12 specific assays for nuclear receptor (NR) and stress response (SR) signaling pathway has been one of the most popular sources for advancing different learning methods such as transfer learning, multitask learning, few-shot learning etc. [9, 18, 19]. Additionally, the PCBA dataset [10] from MoleculeNet and Massively Multitask Learning projects provided more than 120 PubChem bioassays with diverse sets of targets. It consists of curated public datasets, metrics for evaluations, and an open-source library in python called DeepChem [1, 16]. Even though these benchmarks have served to aid cheminformaticians and ML scientists in discovering candidate drugs and allowed for better modeling, their bioassays lack the essential information like disease and target relevance. Benchmarks such as MoleculeNet and ChemProp mostly focus on basic fields like Quantum mechanics or biophysics, but none has focused on specific diseases, the comprehensive class of targets and specially the comparison of these two. The main aim of this article is to connect the biomedical side of the molecular machine learning to the data science side, to infuse the disease classification into training for a real-world purpose. The aim is not to prove that disease categorization is beneficial for every specific type of learning, but to give machine learning scientists the means to model based on disease categorization and to develop better computational technologies with a biological purpose [1, 20].

Annotation and ontology development have been very important sources for scientists to better understand and find the bioassays with unstandardized descriptions in depositories. As an example, Vempati et al. developed a very comprehensive list of classes for a diverse set of variables in bioassays, from diseases to bioassay types [15]. Despite huge contribution to biomedical community to identify the bioassay of interest using specific ontologies, they lack resolution in terms of classes offered for diseases and targets. We believe a reliable and practical ML model can be designed based on a known set of targets for a specific disease, fulfilling the need for a benchmark dataset that provides a comprehensive set of related assays. However, there are major challenges of developing a comprehensive benchmark dataset containing all information such as target classes and disease categories. Firstly, assigning each task to specific disease(s), protein target(s), and signaling pathway(s) categories would be very time consuming, labor intensive, and in some cases, impossible with traditional approaches. Moreover, manually assigning each bioassay to different classes is very error prone. Secondly, clustering such big data requires chemical, biomedical, and computational knowledge which makes it difficult to perform. Therefore, we believe novel technology such as deep learning and natural language processing (NLP) should be employed to confront such challenges of dataset benchmarking. MolData, which is developed by the implementation of novel methods such as NLP, is one of the most comprehensive disease and target-based benchmarks for democratizing molecular machine learning. It consists of 600 diverse bioassays from PubChem which are curated and clustered into 15 different diseases categories, one Toxicity dataset, and 14 unique protein target classes. One of the most important steps in every drug discovery and development process would be molecular toxicity and side effect assessment. Therefore, we decided to have one comprehensive category for Toxicity related tasks due to the importance of it for successful drug discovery. More than 1.4 million distinct molecules are presented in this benchmark, which consists of more than 170 million molecular screening data points. The majorities of the Bioassays (> 85%) include UniProt IDs to assist the extraction of diverse kinds of information including Molecular Function, Biological Process, Pathway Dataset, Protein Family Group, Taxonomy, Subcellular Location, Topology and BLAST, Expression and interactions, etc. MolData aims to assist in the discovery of better and more diverse candidate drugs via the meaningful aggregation of large datasets. In doing so, it can be one of the main sources for the ML and Data Science community to develop practical molecular machine learning models. The main reasons for developing a disease categorized dataset are firstly, to ease the search of ML community and cheminformaticians for datasets of one specific disease or protein targets. Secondly, to help the ML community to develop better technology and modeling not only based on random datasets, but on meaningful disease categorized ones. To demonstrate the application of MolData, we have run a correlation analysis to investigate drug repurposing, from which we have discovered three sets of bioassays highly correlated in both active and inactive molecules. Lastly, we trained more than 30 different multitask learning-based models, each for a specific disease or target, and one for all bioassays combined. These models can serve as a baseline for the data science community in order to advance molecular machine learning and enable better drug discovery.

## Results

### Benchmark creation pipeline

**Fig. 1:**
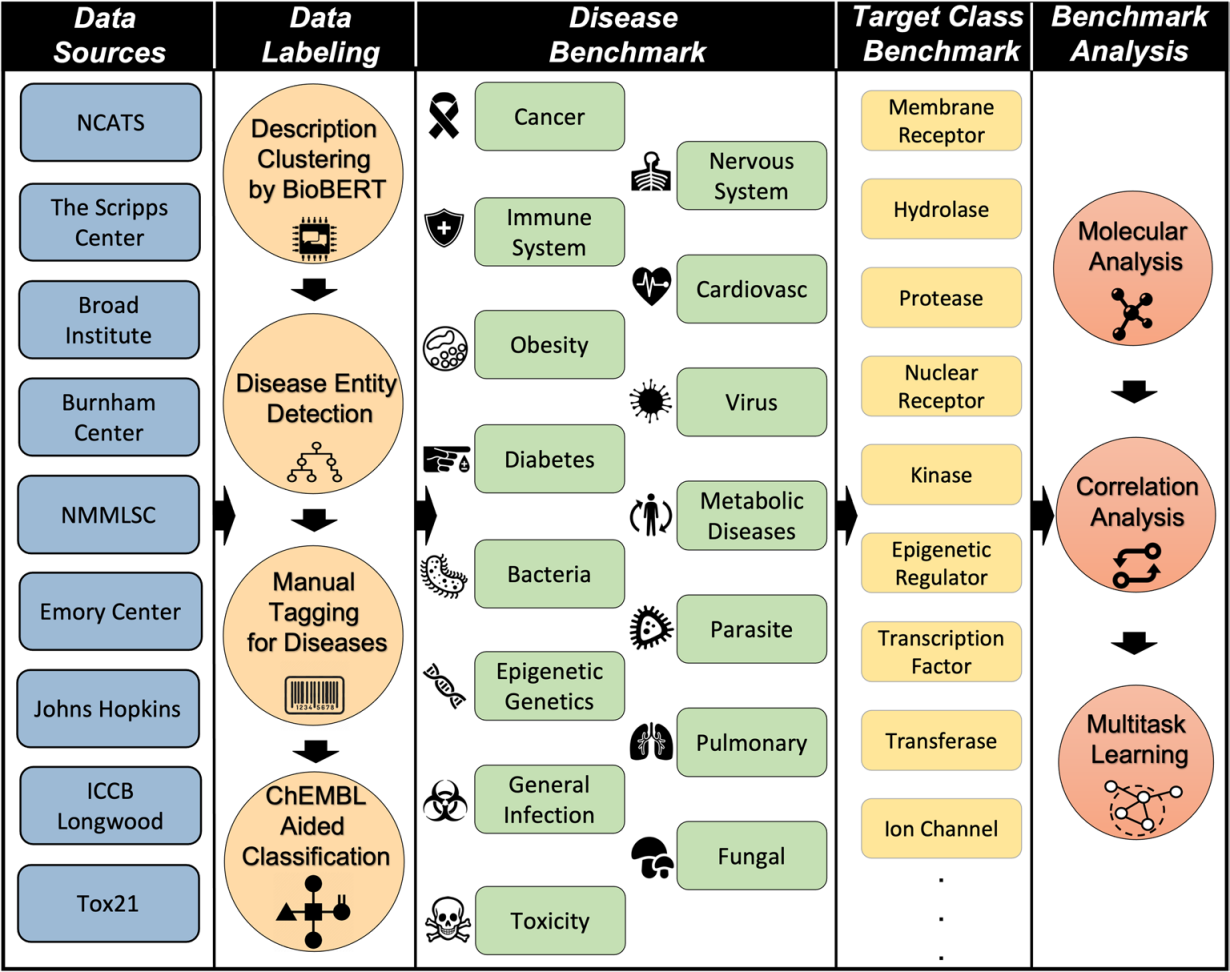
The pipeline of MolData benchmark creation

The overview of the benchmark creation pipeline is depicted in Fig. 1. The process started by downloading the descriptions and summary of each data-source from PubChem. Due to the large number of selected bioassays, computational methods were implemented to aid in the creation of the benchmark and serve as guideline for the manual tagging of each bioassay. The assay descriptions were first grouped into 10 clusters using BioBERT [21, 22], and tagged using a similar disease entity recognition model. Having done so, each description was tagged manually with the assistance of the computational model results. By tagging assays in clusters separately, the similar keywords used for tagging were easier to detect. Manual tagging resulted in sixteen different disease-based categories of data. In addition, we used ChEMBL repository [12] to identify each task’s target class. After assigning each bioassay to one or more disease and target categories, the benchmark was analyzed with multiple approaches. After assigning each bioassay to one or more disease categories using specific keywords, the benchmark was analyzed with multiple approaches, such as mapping the molecular domain. For the application of drug repurposing, we ran a correlation analysis on the data and discovered three sets of correlated bioassays. Finally, different multitask graph convolutional neural networks (GCNNs) [23] were trained in order to create a baseline for the performance of multitask learning models in each disease related category. The main dataset of MolData which is the result of this pipeline can be found on the GitHub repository as well as in Additional file 1.

### Data aggregation results

MolData benchmark originates from 9 open-source data sources on PubChem, which are the largest in terms of number of screened molecules and number of active bioassays [24], as shown in Table 1. Initially, these collected data contained more than 1000 bioassays, which were then triaged to 600 bioassays (Bioassay Identifier will start by “AID” acronym) after filtering datasets smaller than 100,000 molecules or 15 active molecules. We included the updated Tox21 source [25] with more than 55 different bioassays due to their applicability to drug screening. As seen in Table 1, the activity percentage of each screening task was usually less than 1%, showing the imbalanced nature of the screening datasets. The bioassay IDs of the used datasets as well as their related source are available in Additional file 2. All 600 bioassays are cited within an additional reference list included in Additional file 3.

**Table 1:** Data source summary. MolData was created using 9 data sources, the number of bioassays within each data source is shown in AID count column. Each molecule in a given source can have bioactivity for multiple bioassays and constitute multiple data points. Unique active molecules are defined as molecules that demonstrate bioactivity in at least one bioassay

PubChem Source	AID count	Active data points	Total data points	% Active datapoints	Unique active molecules	Total unique molecules	% Unique active molecules
Broad Institute	67	125,627	22.2 m	0.56%	85,579	472,858	18.1%
Burnham Center for Chemical Genomics	67	139,021	21.9 m	0.63%	77,159	381,794	20.21%
Emory University Molecular Libraries Screening Center	12	24,195	2.47 m	0.98%	20,964	348,231	6.02%
ICCB-Longwood Screening Facility, Harvard Medical School	11	8358	2.1 m	0.39%	6656	564,021	1.18%
Johns Hopkins Ion Channel Center	22	48,545	6.8 m	0.71%	35,487	344,497	10.30%
NMMLSC	42	48,186	11.5 m	0.42%	37,949	369,431	10.27%
National Center for Advancing Translational Sciences (NCATS)	174	720,319	53.4 m	1.35%	240,096	592,616	40.51%
The Scripps Research Institute Molecular Screening Center	148	275,224	47.6 m	0.58%	142,055	920,418	15.43%
Tox21	57	21,475	0.47 m	5.67%	4183	8743	47.84%

### Data description domain

**Fig. 2:**
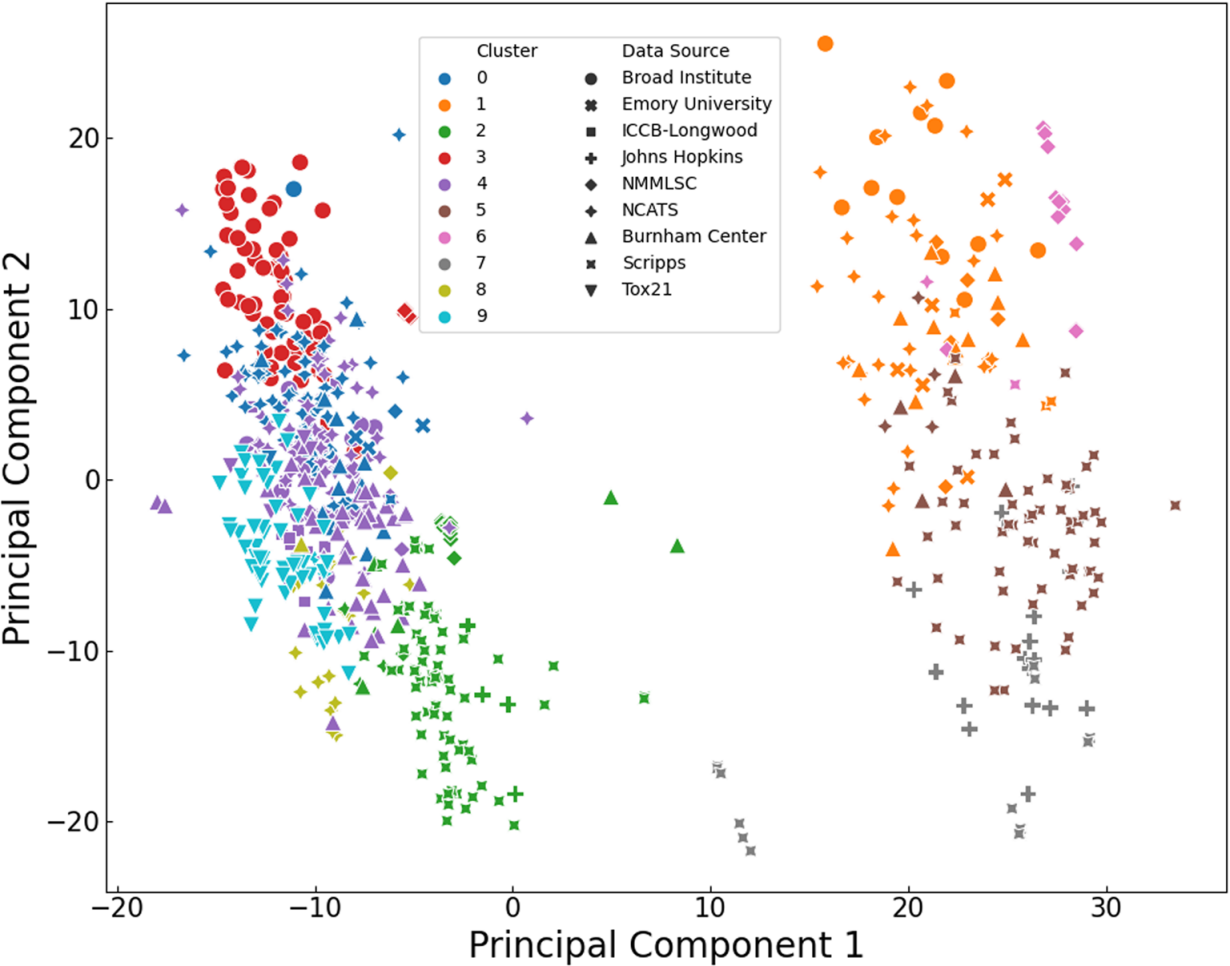
Map of the bioassays’ descriptions using the output of the BioBERT model (The explained variance ratio for the first and second principal components are 14.72% and 5.40% respectively)

To better understand the diversity within the 600 gathered bioassays, the description of each assay was fed to a BioBERT model [21]. BioBERT is an attention-based model, which is trained on a large corpus of biomedical text to predict masked words. This type of training and language modeling gives the model the ability to create meaningful representations from biomedical text inputs which encode the context of the input within fixed size vectors. In this work, BioBERT is used on the description of each assay to extract features and map the domain which these descriptions cover. Figure 2 depicts this map after clustering, showing how descriptions from different sources can have similar context to each other (e.g., bioassays from John Hopkins Ion Channel Center and The Scripps Research Institute Molecular Screening Center in cluster 2) or be distinct from the rest (e.g., bioassays from Tox21 in cluster 9).

The same model trained on disease entity recognition was also used to identify disease related key words in each description [26]. While each cluster had some degree of similarity in terms of the diseases covered within each domain, it was far from perfect in correctly dividing the data domain based on their disease categories. Therefore, manual tagging was performed using the clusters and the disease entities as guidance. This process included highlighting disease related words within each bioassay’s description and using them as tags to represent each bioassay. The dataset descriptions as well as their highlighted words are available in Additional file 2.

### MolData

#### Data summary

After collecting all the specific disease identifiers or key words, we clustered them into 15 different categories. These categories were selected after carefully investigating all disease related words and their counts. The categories are: (1) Cancer, (2) Aging, (3) Bacterial, (4) Viral, (5) Fungal, (6) Parasitic, (7) Cardiovascular, (8) Immunological, (9) Nervous System, (10) Diabetes, (11) Epigenetic and Genetics, (12) Pulmonary, (13) Obesity, (14) Metabolic Disorder, and (15) General Infection. The sixteenth benchmark is for the toxicity of candidate drugs which is a very important part of MolData dataset and the drug discovery process. Since Diabetes is a very important disease involving a large population worldwide [27], we also decided to give Diabetes an independent category even though it should be listed under metabolic disorders. The count of assays for each disease and target categories are shown in Tables 2 and 3, respectively. Overall, MolData consists of 600 bioassays with 1.4 million unique molecules, with nearly half of the molecules possessing activity in at least one bioassay. Moreover, MolData contains 224 tasks belonging to 2 or more disease categories (e.g., a bioassay that relates to both cancer and immune system). The MolData benchmark data is available at https://GitHub.com/Transilico/MolData. All molecules, binary labels and splits are available in one file (Additional file 1), with two mapping files containing the mapping of each bioassay to each disease category and to each target category. Overall, MolData offers two benchmark categories: disease categories (as well as toxicity) and target categories, and using these mapping files the molecular data can be selected to create each category.

**Table 2:** Disease-based information for the MolData Benchmark

Tag	AID count	Active data points	Total data points	% Active datapoints	Unique active molecules	Total unique molecules	% Unique active molecules
All Categories	600	1,410,950	168,345,532	0.84	672,935	1,429,989	47.06
Cancer	236	575,454	68,649,771	0.84	230,049	1,323,311	17.38
Nervous System	174	378,812	54,753,975	0.69	170,353	651,249	26.16
Immune system	129	322,362	38,418,661	0.84	157,333	579,658	27.14
Cardiovascular	94	212,162	28,660,627	0.74	124,270	542,902	22.89
Toxicity	54	48,653	2,452,656	1.98	30,936	487,219	6.35
Obesity	53	90,837	14,516,199	0.63	65,993	545,513	12.1
Virus	47	113,946	14,679,312	0.78	81,702	621,945	13.14
Diabetes	43	61,408	11,645,151	0.53	47,830	543,600	8.8
Metabolic Disorders	42	126,772	9,985,491	1.27	70,665	527,382	13.4
Bacteria	40	132,593	12,314,737	1.08	89,554	1,290,782	6.94
Parasite	24	98,950	7,302,206	1.36	75,027	500,228	15
Epigenetics, Genetics	23	92,837	6,815,597	1.36	65,244	439,537	14.84
Pulmonary	19	45,940	6,122,297	0.75	36,467	524,167	6.96
Infection	11	93,444	3,312,920	2.82	63,782	521,473	12.23
Aging	10	9030	3,079,580	0.29	8527	511,471	1.67
Fungal	7	9253	2,147,751	0.43	8824	444,373	1.99

**Table 3:** Target-based information for the MolData Benchmark

Target	AID count	Unique target count	Active data points	Total data points	% Active datapoints	Unique active molecules	Total unique molecules	% Unique active molecules
All Targets	383	296	862,370	103,440,515	0.83	261,715	675,161	38.76
Membrane receptor	85	44	146,956	25,922,533	0.56	91,489	458,818	19.94
Enzyme (other)	54	51	83,657	16,210,090	0.51	57,808	632,142	9.14
Nuclear receptor	53	25	74,776	6,083,509	1.22	42,838	442,487	9.68
Hydrolase	36	32	113,185	10,830,324	1.05	66,195	526,391	12.57
Protease	29	26	37,943	7,965,313	0.47	30,619	606,793	5.05
Transcription factor	27	18	53,416	4,775,685	1.11	40,067	503,249	7.96
Kinase	24	23	38,257	7,369,690	0.52	31,327	377,519	8.29
Epigenetic regulator	23	20	76,793	6,840,095	1.12	51,776	523,904	9.88
Ion channel	22	14	37,402	6,745,762	0.55	28,853	511,873	5.63
Transferase	18	17	43,955	6,279,651	0.7	30,432	519,646	5.85
Oxidoreductase	10	8	33,956	2,953,760	1.15	30,054	432,578	6.94
Transporter	9	8	15,390	2,538,579	0.60	15,046	369,621	4.07
NTPase	6	5	114,465	1,981,575	5.78	76,334	439,967	17.34
Phosphatase	5	5	8090	1,693,773	0.48	6913	368,329	1.87

**Fig. 3:**
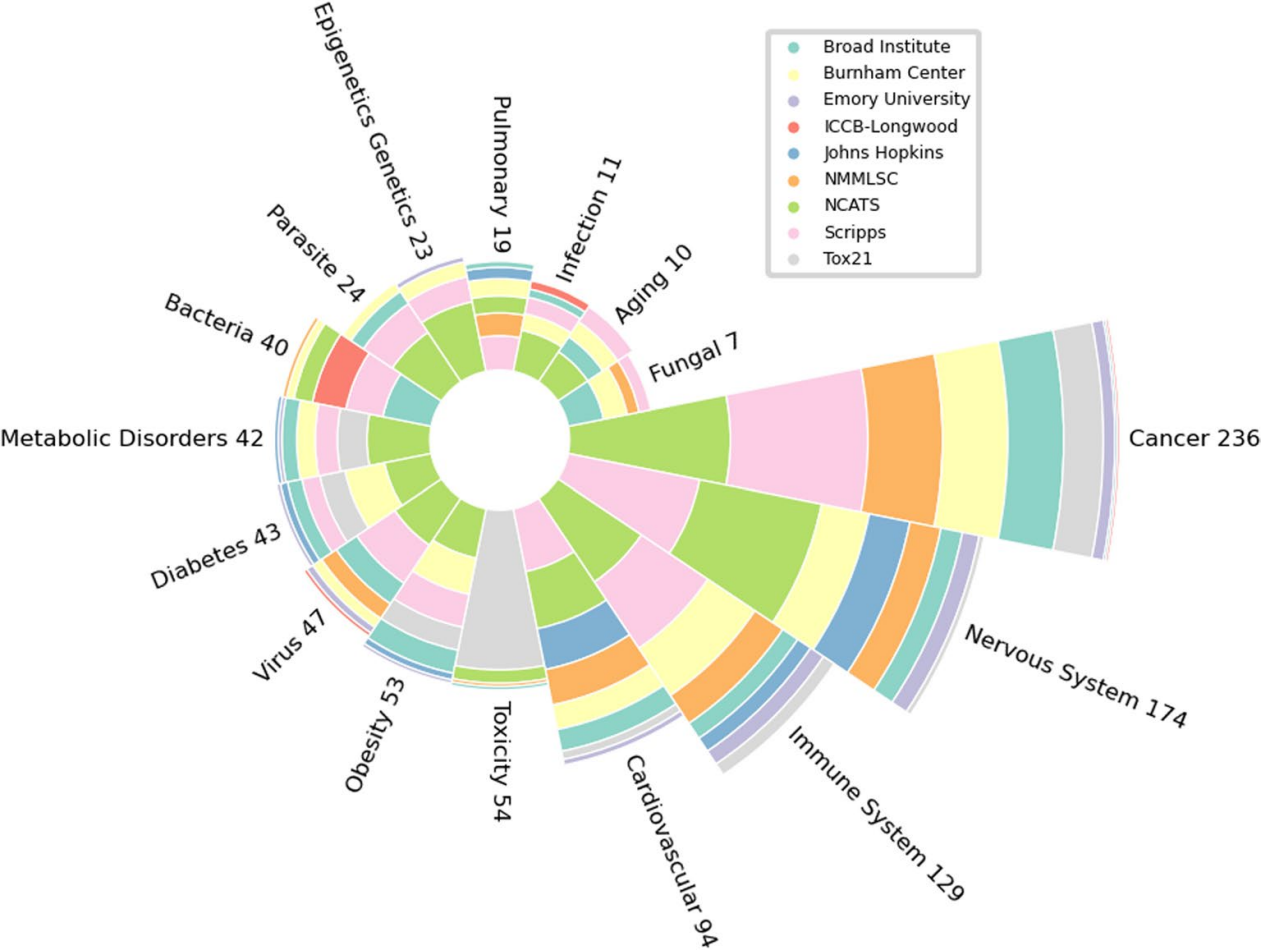
The composition of the disease benchmark: Number of bioassays for each disease category (as well as toxicity) and their original source

The composition of each data category is depicted in Fig. 3; showing how combining data from each data source resulted in the creation of each category. This combination demonstrates one of the main motivations for this work’s data aggregation, as each disease category has related bioassays with multiple data sources. Furthermore, some categories such as Aging and Pulmonary are unexplored compared to those like Cancer and Nervous System, when large screening data is examined. These categories were selected based on their importance and the number of occurrences.

**Fig. 4:**
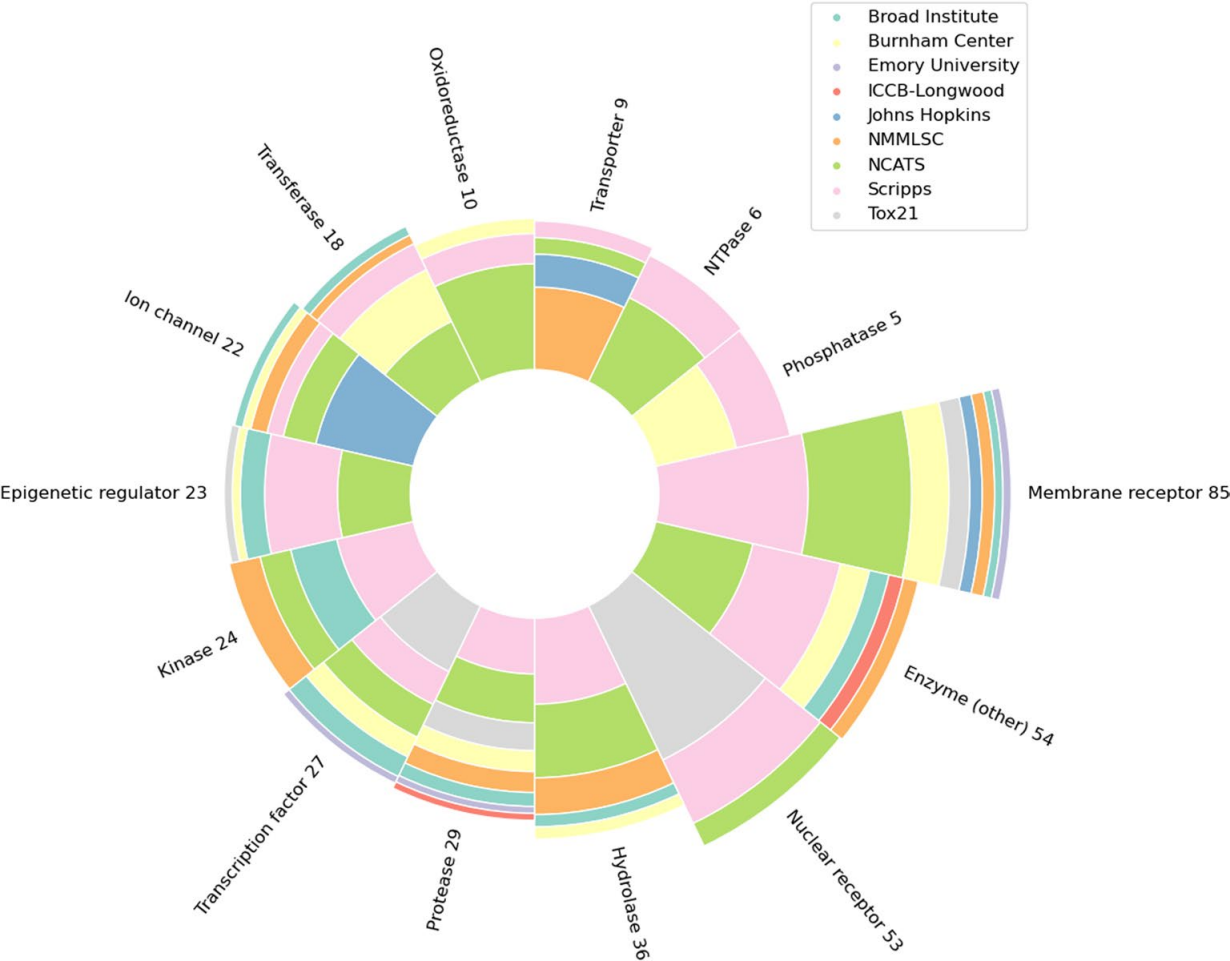
The composition of the target benchmark: Number of bioassays for each target category and their original source

The protein targets of MolData in Fig. 4 were classified by either (1) direct mapping to the ChEMBL database, (2) finding highly similar target in ChEMBL, or (3) manual curation (see “Methods” section). From the 419 total unique targets in MolData, 296 were classified into 14 classes (Fig. 4). Enzymes (167/296) (Enzyme (other) + Hydrolase + Protease + Kinase + Transferase + oxidoreductase + NTPase + phosphatase) are the most prevalent class, followed by membrane receptors (44/296) and nuclear receptors (25/296). The occupancy of target classes is also reflected in the total assays for each class. For example, enzymes constitute the most prevalent class among the targeted assays (182/383), followed by membrane receptors (85/383) and nuclear receptors (53/383). The assays are overall enriched in the “privileged” targets, that is, membrane receptors, kinases, nuclear receptors, and ion channels. These four classes have been historically the most prevalent among approved drug targets [28], accounting for 70% of the total approved drugs. In our dataset, however, 199 assays (52% total) represent targets from classes other than membrane receptors, kinases, nuclear receptors, and ion channels. When counting the total unique targets, these historically “unprivileged” targets even give a higher representation of the dataset with 190 counts (64% total). Therefore, MolData captures a higher diversity in the target classes compared to those of the approved drugs. There are 515 tasks with PubChem designated targets within the 600 tasks. This work was able to categorize 383 of those tasks into specific target categories that have 5 or more bioassays within each category.

There are, additionally, classes that are overrepresented by our dataset compared to the set of targets with available approved drugs. For example, NTPases are targeted by 76,334 unique compounds (29% of the total compounds from targeted assays), while only 2% of drugs target NTPases. Additionally, epigenetic regulators represent the target of 51,776 unique compounds (20% of the total compounds from targeted assays), while only 0.3% of drugs interact with this class of proteins [28]. These higher hit rate in the targets of MolData compared to the approved drugs could imply the inherent low druggability of such target classes or the lower significance of the targets for pharmaceutical industries.

#### Molecular domain

**Fig. 5:**
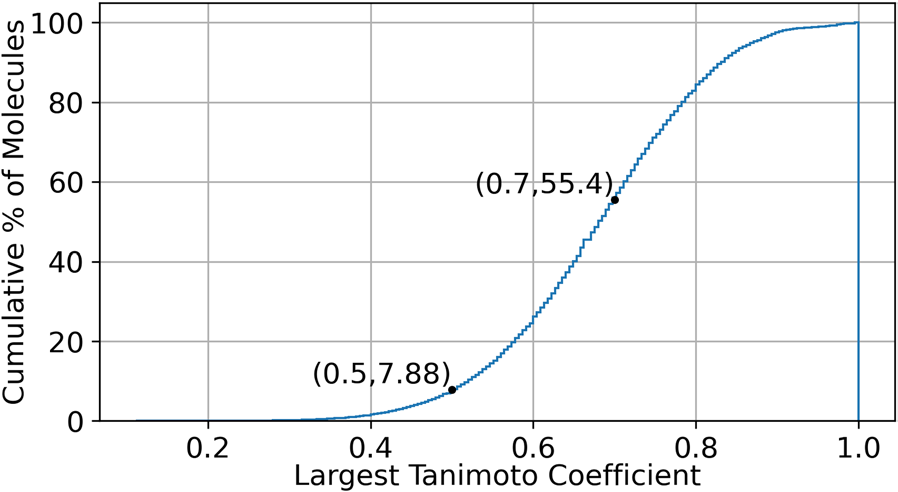
Cumulative histogram of the largest Tanimoto Similarity Coefficient for 200,000 molecules within the MolData dataset. More than 92% of the molecules have other similar molecules to them within the dataset with Tanimoto Coefficient of higher than 0.5, and more than 44% of the molecules for Coefficient of 0.7

To investigate the diversity of the screened molecules, all collected molecules are represented as vectors using ECFP4 [26]. This binary fingerprint of size 1024 encodes the existence of sub-structures within the molecules using ones and zeros. Similarity between the fingerprint of two molecules can be calculated using the Tanimoto coefficient, where the overlap between the fingerprints is divided by the union of the fingerprints. This similarity coefficient can be used to find the similarity between all molecules within a dataset to assess the diversity of a molecular dataset [30, 31]. The cumulative histogram for maximum similarity coefficient for a random selection of 200,000 molecules within MolData is shown in Fig. 5.

Figure 5 demonstrates that more than 44% of the molecules within the MolData dataset have at least one other similar molecule to them with a Tanimoto Coefficient of 0.7 or higher. This high percentage of the similarity can denote lack of diversity within this portion of the dataset and may be result of existing predefined rules within the tradition molecule selection techniques for drug candidate screening. The effect of this lack of diversity is discussed as bias within the discussion section.

#### Correlation analysis, a showcase for drug repurposing

Drug repurposing is the process of finding new applications for already approved molecular drugs. These new applications can be target or disease based depending on the specific case of study. For example, during an outbreak, drug repurposing could be the fastest and most efficient option due to a lack of information about the new virus/bacteria. [32]. Azithromycin, a macrocyclic antibacterial, has shown to be effective against Ebola virus with EC_50_ of 5.1 $\upmu$M [33]. It also has shown promising results as a potential antimalarial (*Plasmodium falciparum*) when prescribing alone or in combination therapy [34–36]. For this benchmark, we hypothesized that correlating bioassays screened on different sets of targets would provide interesting information for better and faster drug repurposing. Therefore, the correlation score between the molecule bioactivity labels were calculated using a Pearson correlation coefficient.

**Fig. 6:**
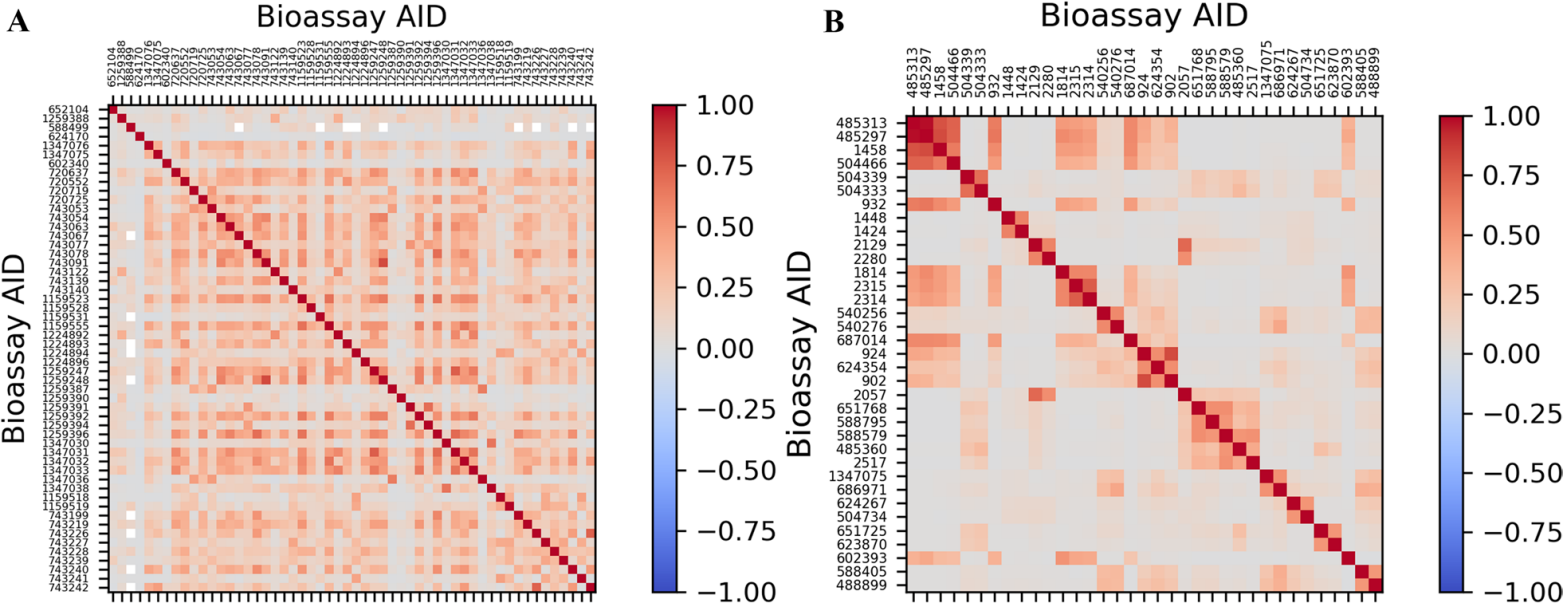
Correlation matrix between **a** Toxicity bioassays, **b** Non-Toxic bioassays with correlation > 0.5

Between all categories, toxicity showed the highest correlation of tasks, which is understandable due the nature of toxicity and the close biological relationship between the assays. In Fig. 6, correlation heatmaps are shown for Toxicity assays and all non-toxicity assays with a correlation of 0.5 or more which have different targets. The second chosen group indicates higher correlation can exist between the labels of bioassays from the same, or different sources. Two sets of correlating targets and a viral similarity were discovered through this analysis. The first set of targets with a high correlation were (1) NPC1 (2) SMN1 (3) ATAD5 (4) Rab9 (5) STAT1. NPC1 and Rab9, with a 98% correlation, are important players in cholesterol metabolism and Niemann Pick Disease Type C (Additional file 4) [37, 38]. AIDs 485,297 and 485,313 were designed to discover the activators of mentioned proteins using luciferase reporter assays. Their high correlation to assays targeting STAT1 or ATAD5, which are important in cancer and immune disorders [39–41], is a valuable finding by a simple linear correlation analysis of MolData benchmark for drug repurposing. Another interesting discovery was infectious disease based, as molecules targeting the Lassa Virus and Marburg Virus showed a high correlation. The Lassa Virus is a single stranded RNA virus with a circular morphology from the family of Arenaviridae, and is cause of Lassa hemorrhagic fever [42, 43]. Moreover, the Marburg virus belongs to the family of Filoviridae, with a shepherd's crook morphology, and causes similar symptoms to the Ebola virus, with a fatality rate of ~ 50% [44, 45]. Both bioassays used the viruses envelop glycoproteins on a pseudotype virus system. We were curious to see if there has been any candidate drug with promising potency against both viruses. Favipiravir is a pyrazine carboxamide derivative that has shown effectiveness against both the Lassa and Marburg viruses [46, 47]. These data suggest that MolData would be valuable source for further drug repurposing investigations. We suggest the further investigation of MolData correlation analysis using experimental assays to better understand the findings of this paper.

#### Benchmark classification modeling, a showcase for bioactivity prediction

The data from each disease and target category, as well as the aggregation of all bioassays, are used as training inputs for GCNNs. The classification results are shown in Tables 4 and 5 as the baseline for each category. These results are from the imbalanced (untransformed) test sets and validation sets, weighted to ignore missing data points for each task (weight of 0), then averaged across all tasks within each category. This weighted evaluation on untransformed data with missing values is made accessible in “training” script on the GitHub repository. The detailed results for each model and bioassay are presented in Additional file 5. These results show the baseline performance for multitask models, with ROC AUC serving as the most important comparison metric due to the imbalance nature of the data.

**Table 4:** Classification results on the validation and test sets of disease categories, averaged on all tasks within each category

Benchmark	Validation Set				Test Set			
	Accuracy (%)	Recall (%)	Precision (%)	ROC AUC	Accuracy (%)	Recall (%)	Precision (%)	ROC AUC
All Tasks	64.7	76	3.93	0.7803	63.96	75.69	3.98	0.774
Cancer	73.61	68.76	3.68	0.7809	72.96	68.44	3.76	0.7765
Nervous System	73.34	65.1	2.39	0.7573	73.01	64.92	2.49	0.7556
Immune System	79.7	61.34	3.41	0.777	79.49	61.01	3.5	0.7739
Cardiovascular	80.06	56.84	2.98	0.7498	80.06	56.39	3.13	0.7457
Toxicity	86.9	33.41	24.46	0.7445	86.51	34.27	27.54	0.7309
Obesity	86.01	54.1	5.42	0.7925	85.37	51.5	5.51	0.7704
Virus	77.73	62.08	2.62	0.7625	77.9	61.91	2.84	0.7643
Diabetes	86.69	51.27	5.8	0.7845	85.88	51.33	5.99	0.7795
Metabolic Disorders	83.14	53.04	6.89	0.7619	82.71	54.85	7.06	0.7619
Bacteria	83.1	60.82	4.63	0.7916	82.26	64.49	4.69	0.8089
Parasite	91.51	46.65	11.31	0.8292	91.37	44.63	11.17	0.8243
Epigenetics-Genetics	88.46	45.27	6.36	0.7804	88.32	40.98	5.65	0.7251
Pulmonary	76.82	56.7	2.34	0.7293	76.06	54.79	2.5	0.7168
Infection	92.17	31.58	12.53	0.801	92.01	29.87	11.41	0.7871
Aging	94.83	23.59	1.86	0.7205	94.28	29.36	2.38	0.7402
Fungal	92.36	35.22	3.5	0.75	92.77	33.93	3.61	0.7335

**Table 5:** Classification results on the validation and test sets of target categories, averaged on all tasks within each category

Target Benchmark	Validation set				Test set			
	Accuracy (%)	Recall (%)	Precision (%)	ROC AUC	Accuracy (%)	Recall (%)	Precision (%)	ROC AUC
All Tasks w/Targets	73.89	69.7	4.81	0.789	73.54	68.6	4.86	0.7786
Membrane receptor	79.67	56.29	2.45	0.7479	79.77	53.54	2.44	0.7251
Enzyme (other)	85.32	60.81	2.86	0.8123	85.05	60.2	2.98	0.8019
Nuclear receptor	84.45	47.15	16.98	0.7516	84.95	45.96	18.23	0.7511
Hydrolase	84.35	65.1	3.35	0.8165	84.09	59.5	3.43	0.7879
Protease	85.88	51.43	3.9	0.7874	85.21	52.53	3.89	0.7792
Transcription factor	86.72	49.45	12.59	0.7947	85.35	51.9	13.37	0.7858
Kinase	77.56	51.29	2.34	0.7142	77.53	49.77	2.33	0.6955
Epigenetic regulator	86.22	58.25	6.12	0.8097	85.55	55.25	6.23	0.8077
Ion channel	96.82	22.63	6.81	0.7496	96.68	21.13	6.16	0.7393
Transferase	93.32	45.94	8.43	0.837	92.86	42.59	7.75	0.8234
Oxidoreductase	93.25	25.42	19.1	0.7977	92.77	26.4	11.27	0.7994
Transporter	94.93	19.21	3.67	0.7008	94.86	16.88	3.97	0.663
NTPase	93.68	10.45	41.43	0.7479	93.33	9.59	26.78	0.7639
Phosphatase	98.53	21.86	15.27	0.7993	98.45	19.13	14.48	0.815

While the results differ between all benchmark categories, in general the models have higher recall and lower precision. Further interpretations of these results as well as the next steps for improving model performance is discussed within the discussion section.

Moreover, in conjunction to the GCNN model, to further investigate the values of the baseline and to better evaluate the results of the benchmark on traditional virtual screening models, a simple fully connected neural network model was trained on the Extended Connectivity Fingerprint (ECFP) features of the molecules. The results of this neural network model can be viewed in Additional file 6, where all benchmark results follow the trend of the GCNN, with slightly lower values across the tables, demonstrating that GCNN was able to give better results that the fully connected neural network.

#### Use cases of MolData

The current available assays within the PubChem database include detailed biological descriptions on the purpose of the assays and the specific question they investigate. While this is essential and useful for experts within the field of biomedical sciences, the narrow scope of these questions makes it hard for the machine learning and data scientists to pursue modeling the available dataset. The description of public dataset often contains extensive information about their cellular target, the final purpose of the assay, applications of the assay for specific disease(s). Therefore, it would be very hard to completely understand all important information in such dataset by scientists without biomedical background. In this work, these datasets with narrow scopes are categorized in ways that would give a wider scope to what they are investigating (e.g. disease or target categories) and would define a more tangible purpose for the in-silico drug discovery community to pursue.

From a biological point of view, MolData can be used to offer a large list of datasets, where each dataset is tagged and labeled to be related to a disease. Given the interest of the user, MolData datasets can be examined and selected for further research in two manners. The user can either create models using the offered dataset, or augment the related datasets within MolData to other data to increase the scope of modeling or to investigate new questions of interest that arise from the diversity of datasets within each disease category.

From a machine learning point of view, MolData offers three use cases: firstly, a molecular database with 1.4 million molecules and 600 possible labels, where each label relates to a disease category. This dataset proposes a challenge to the machine learning community due to its size and its imbalanced nature and can be of interest to the in-silico drug screening community due to its composition of completely disease related tasks. Secondly, MolData offers individual benchmarks with biological purposes, where the categories are designed to have tangible objectives such as diseases and targets. Therefore, any improvement within the benchmark values can be associated with impact on the early drug discovery stage for those diseases and targets. And lastly, MolData can be combined with other datasets to investigate the effects of multitask learning when multiple tasks from a disease category or multiple tasks from a target category are used for training. This last use case is further investigated in Additional file 6.

## Discussion

Overall, the pipeline implemented in the creation of MolData resulted in aggregation of molecules from 9 large data sources (Table 1) with 600 bioassays that have a diverse set of descriptions (Fig. 2). Even though these descriptions have a wide range, different data sources can have similar descriptions (Fig. 2), which encourages combining the data from these sources. However, the BioBERT clusterization of bioassays did not provide an accurate disease or target related label in most cases, due to the existence of disease related words (i.e., cancer) in many bioassay descriptions that did not relate to that disease. To overcome this problem and to present accurate tagging, BioBERT clusters were only taken as recommendation and manual tagging was performed, where highlighted keywords were used to categorize bioassays into disease and target related categories. The resulting dataset consisted of bioassays covering multiple diseases and targets (Figs. 3, 4) which gave the opportunity to create multiple benchmarks with biological purpose.

One of the main topics worth discussing is bias within the dataset. MolData consists of roughly 170 million data points. However, this screening was performed on 1.4 million molecules, denoting that each molecule exists on average in nearly 117 assays. Since the data sources are different, this level of repetitiveness shows a large overlap of molecules within the original data sources. Furthermore, as seen from the results of the molecular domain analysis in Fig. 5, many of the molecules are also similar to each other within the dataset. Therefore, a degree of bias exists within the gathered dataset with similar molecules being screened for each assay and in all data sources. We speculate this bias is due to the traditional rules used for selecting molecules as candidates for screening.

One effective way to increase the diversity of chemicals would be switching from screening synthetic libraries to natural product libraries. Natural derived compounds have shown a higher hit rate with the potential of targeting unknown and complex biotargets [48, 49]. The first reason why natural product-based molecules show better hit rate and potency would be their complexity and structural diversity [49, 50]. Secondly, these molecules have helped live organism to battle microbial invasions during millions of years of evolution which have made them better over time [51, 52]. However, there have been major challenges with their discovery and the use of natural products. Since most of these natural product’s libraries are discovered from natural organisms and animals, their extraction imposes a threat to many species and increase the risk of their extinction. Also, their discoveries are more tedious and expensive than just synthesizing non diverse organic molecules in labs. At the end, synthesizing some of nature driven molecules are very hard and sometimes impossible. Finally, since their major roles in microorganisms have been as a defense line, they would show activity against multiple bio-targets in a cell which increase the probability of showing toxicity in drug development [49, 50, 52]. Therefore, there has not been practiced in HTS assays like simple synthesized molecules. However, learning methods like GCNN has shown potentials in discovering them even with no nature driven molecular data when training [35].

Another important topic to consider is the benchmark modeling result. The model architecture was selected to be shared within all models; however, this is suboptimal, and hyper-parameter optimization can be performed to find better possible architectures for each data category. This can apply to other hyper-parameters such as learning rate and batch size, which can be improved via a grid-search hyper-parameter optimization. Lastly, the low precision of the models is a focus of improvement since precision plays an important role in selecting molecules for future screening at inference time, directly affecting the cost and time of screening.

## Conclusion

MolData is one of the largest efforts in the collection, curation, and categorization of labeled molecular datasets. It consists of roughly 170 million screens of 1.4 million unique molecules distributed in 600 different bioassays and 15 disease categories and one toxicity category, from cancer to infectious diseases. It also consists of a state-of-the-art target benchmark with 14 categories. We explored all the disease and target-related details in each bioassay for the development of a comprehensive benchmark to assist data scientists and the ML community in improving model development and computational drug discovery with benchmarks that have tangible biological purposes. We believe a key feature of any learning system is the training data, and the validation of a model is only possible with appropriate molecular and biological knowledge of the dataset. MolData takes advantage of a greater amount of labeled data compared to other benchmark datasets, which is an important addition to CADD. It is beneficial for the data science community to have a similar dataset for comparison of model performances; therefore, baseline performance is presented for 32 different categories. MolData hopes to take a step in furthering the molecular machine learning revolution, by providing the means for drug discovery and model development with a biological purpose.

## Methods

### Data aggregation

The dataset was collected from PubChem bioassays due to its comprehensiveness and the high diversity of diseases and targets. We started with the selection of PubChem sources with highest number of Live Bioassays Counts, High Throughput Screening (HTS) capabilities, and being open-source. Hence, we selected nine sources as follows:

1. National Center for Advancing Translational Sciences (NCATS) is one of the most comprehensive centers for drug screening with a goal of therapeutic development trough collaborative research [5, 10],
2. Broad Institute of Harvard and MIT with a focus on assay development and scientific collaboration for the advancement of Discovery Science and Translational Pharmacology. They have the capability of screening 100 s to 1,000 s of compound plates a day [54, 55],
3. Sanford-Burnham Center for Chemical Genomics is a well stablished screening center working on multiple projects including NIH Molecular Libraries program (MLP) with applications on multiple diseases [56, 57],
4. NMMLSC is an screening center with capability of using high throughput flow cytometry to discover molecules as chemical probes for drug discovery [58, 59],
5. Emory University Molecular Libraries Screening Center with focus on Biological Discovery through Chemical Innovation and also molecular pathogenesis to global pandemics. [60, 61],
6. Tox21 which contains thousands of medicinal or environmental substances which is a collaboration between NCATS and national toxicology program. Tox21 is an ongoing project with yearly update [62, 63],
7. The Scripps Research Institute Molecular Screening Center is an automated center with projects on a variety of diseases like Alzheimer and cancer. They also have capability of assay development, Compound synthesis cheminformatics, mechanism of action discovery etc. [64, 65],
8. Johns Hopkins Ion Channel Center with a focus on membrane proteins and transporters which are permeable to ions [67]. Due to the importance of this class of targets, we decided to include them as one of main sources.
9. ICCB-Longwood Screening Facility, Harvard Medical School which performs most of the HTS assays with the availability of over 500,000 molecules for screening [68, 69].

Aforementioned sources were also selected due to their credibility of HTS data. As the final goal of this article is providing the machine learning community with a large, clustered dataset, we decided to include bioassays containing 100,000 or more molecules screened, as well as bioassays with more than 15 unique active molecules. This threshold was not applied to the Tox21 assays, which have a lower number of screened molecules, which were selected due to the importance of toxicity prediction to drug discovery. Table 1 shows the exact number of each sources’ count, as well as active/inactive molecules. To collect data from each source, the PubChem website interface was used, where data sources were sorted by their bioassay count. The same interface also offers the ability to download the molecular data as well as the summary (and descriptions) of all bioassays within each source.

### Mapping the data domain with natural language processing

After the assays are gathered and filtered by a size threshold, the process of understanding the context of the assays begins. Each assay contains information including the title of the assay, a general description, and optionally the biological target of the screening. To understand the diversity of the assays and map the domains which they cover, the description of each assay is analyzed using natural language processing tools, as elaborated upon in the following subsections.

#### Description pre-processing

The description of each bioassay was acquired from the PubChem website. Each description can contain a complete molecular and biological background, goal of each assay, and finally a brief description of the biological assay. However, each description may also contain unusable information such as the affiliated center, references, scientists involved in the screening, and grant information. Using Python string parsing capabilities, manual rules were written for of each of the eight data sources to filter out the lines containing the unusable information, resulting in cleaned descriptions explaining the assays’ goal. These rules can include deletion of lines pertaining to Principal Investigators, grant numbers, Screening Center Affiliation, Network, Assay provider, Grant Proposal Number, etc. from the description to extract only the assay description from the text. Description preprocessing is made accessible in “preprocessing” script on the GitHub repository.

#### Feature extraction using BioBERT

Feature extraction from a piece of text is typically done via a model which is capable of taking a varied length piece of text and turn it into a fixed-length numerical vector. In the biomedical processing domain, this is typically done via Word2Vec [70] models or language models such as ELMO [71] or BERT [72]. The descriptions that are found for each bioassay typically contain many biologically relative information and words, which the normal language models are not suitable to handle. Therefore, a language model is needed which is trained on biological text and has the related words within its vocabularies. BioBERT is a bidirectional transformer model constructed of multi-head attention modules. This model is trained for language modeling on a plethora of biomedical literature, predicting the masked tokens from raw unlabeled text. Using this pre-training, the model can generate meaningful representation from biomedical text and encode the input in a discernible feature vector. In this work, The cleaned descriptions were lower-cased and fed to a BioBERT model for feature extraction. Leveraging the feature extraction capability of this model, each description is transformed to a numerical vector of size 2048, representing what each assay’s description contained. One disadvantage of this technique is the limited input size of BioBERT (512 token), which resulted in truncation of some of the descriptions. This process is repeated for the titles of each bioassay as well, resulting in another vector of size 2048, which was concatenated with description’s vector. With the rule-based cleaning of the descriptions as described in previous subsection, the inputs of the model were cleaned to be the description itself, to include the most information possible within the 512 tokens. Moreover, the title of the bioassay was added to the beginning of the description to always be included within the input of the model. It is noteworthy that the descriptions examined for manual tagging were the full untruncated descriptions, and since BioBERT is used just as a tag recommender, this truncation does not affect the final output of the manual tagging. Feature extraction using BioBERT is made accessible in “clustering” script on the GitHub repository.

#### Clustering

Having acquired feature vectors of assay descriptions and titles, they are clustered using K-Means clustering. Since the target of this clustering is to explore the domain which the descriptions cover, the number of clusters are unknown. To find the optimum number of clusters, the sum of squared distances of data points to their closest cluster center (SSE) are calculated and plotted based on the number of clusters. The optimum number of clusters is then found by detecting the knee point of the plot. Clustering the descriptions using KMeans is made accessible in “clustering” script on the GitHub repository.

### Tagging the assays

After distinct clusters are formed from assay descriptions and the domains covered by the datasets are better defined, different assays can be grouped together to form a benchmark. The main form of distinction between the assays chosen in this work is disease category relations. As previously mentioned, it is important for a dataset to provide each bioassay with simple disease and target categories for better computational drug discovery. To find the related disease categories for each assay, the process of tagging is used, during which certain words in the description are chosen as tags to represent the assay. This process was implemented both using AI assistance and manual annotation.

#### BioBERT disease category entity recognition

The first approach implemented in this work to extract the disease related words from the description text of an assay is using a BioBERT model trained for disease category entity recognition. This model takes a text sequence as input and returns the entity class related to each token, with the classes consisting of disease and non-disease category entities. Using this model, all related disease keywords are extracted from each assay, automating the process of tagging. The script for disease category entity recognition is provided as “ner” script within the GitHub repository. However, one major disadvantage of this technique is that many words within the description are disease category related, but not defining for that assay. As an example, a task would claim that an older drug for a specific virus would be a carcinogen, falsely adding a disease tag related to “cancer” to the assay. The mentioned assay would have nothing to do with cancer and was just an effort for antiviral drug discovery.

#### Manual tagging

Since many of descriptions contain some biomedical-related words that are not defined for that specific task, understanding the exact biological assay and diseases related to the screening are crucial for tagging. A bioassay description contains a large amount of information regarding the target, related disease categories, other proteins/RNAs/DNA down or up-stream, and in some cases the experimental details of the bioassay. In a task description below, we provide a description from BioBERT cluster zero for AID 1,259,313 from Burnham Center for Chemical Genomics entitled “uHTS identification of small molecule modulators of NR3A”. As shown in this figure, we first read the description for better understanding the assay as a whole, as well as the tags found by the computational method, and then highlight any words with the potential of directing us to a special disease category. Here, Central Nervous System (CNS), Down Syndrome, and Neurological Disorders are the main words that direct us to the subcategories of ‘Nervous System’ and ‘Epigenetics-Genetics’. Reading manual tags from a word file is provided in the “manual_tag” script within the GitHub Repository.

**Table:** 

Activity of N-methyl-D-aspartate subtype of glutamate receptor (NMDAR) is essential for normal central nervous system (CNS) function. However, excessive activation of NMDAR mediates, at least in part, neuronal or synaptic damage in many neurological disorders, including hypoxic-ischemic brain injury and in Down syndrome. The dual role of NMDARs in normal and abnormal CNS function imposes important constraints on possible therapeutic strategies aimed at ameliorating or abating developmental disorders and neurological disease: blockade of excessive NMDAR activity must be achieved without interference with its normal function. We propose an approach for NMDAR modulation via modulation of the NR3A subunit, a representative of a novel family of NMDAR subunits with the goal to modulate the NMDAR activity. NR3 subunits have a unique structure in their M3 domain forming part of the channel region that contributes to decreased magnesium sensitivity and calcium permeability of NMDARs. It potently and specifically binds glycine and D-serine, but not glutamate. In addition, we have shown that glycine binding to the ligand-binding domain (LBD) of NR3A is essential for NR1/NR3 receptor activation, as opposed to internalization caused by ligand binding to NR1 LBD.	

### Benchmark creation

After the disease related words are highlighted and extracted, each assay can be represented by its tags. The next step of the process is to use these tags for grouping related assays together, and to create the benchmark. To do so, major disease categories were first identified which could encompass all tags; and second, each tag was assigned to one or more related major disease categories. The relation between each tag and the major disease category can be found in Additional file 7.

The classification of the protein targets of our dataset was gleaned by downloading and searching against the ChEMBL 29 [12] database (https://ftp.ebi.ac.uk/pub/databases/chembl/ChEMBLdb/latest/chembl_29.fa.gz). If the sequence of a given target—retrieved from UniProtKB using its UniProt ID matched identically to that of a ChEMBL target, the target classification was copied from ChEMBL. To classify the targets missing in ChEMBL, an all-by-all pairwise alignment was performed between MolBio targets and ChEMBL 29 dataset using phmmer 3.3 [73]. If the top-scoring phmmer hit from ChEMBL aligns to the query sequence with a bit score of at least 100 and shares more than 80% similarity in sequence length, the classification is copied from the ChEMBL hit. The targets that neither mapped to ChEMBL nor aligned confidently to ChEMBL using the mentioned criteria were annotated manually. The dataset originally contained 17 classes, but the list was curtailed to 13 classes to remove the ones with assay occupancy of fewer than 5.

#### Molecular data pre-processing

Having populated the disease categories, the molecular data for each assay was downloaded in the form of Simplified Molecular-Input Line-Entry System (SMILES) and their related bioactivity [74]. To curate the SMILES, During this step, the SMILES is first transformed into a Mol Object, then reverted back to a canonical SMILES format with isomeric information. The SMILES input for each molecule was canonicalized with isomeric information included using RDKit version 2020.09.1. Duplicate or missing SMILES entries were then deleted using Python 3.6 and Pandas library version 1.1.5. Regarding the bioactivity of the molecules, the existing labels in all assays are “Active”, “Inactive”, “Inconclusive”, and “Unspecified”. For the sake of consistency, molecules with inconclusive and unspecified labels were removed, and active and inactive molecules were respectively labeled as 1 and 0. Having cleaned the datasets, those bioassays with less than 15 active molecules were deleted, since they would offer a challenge during data splitting. Molecular data preprocessing is made available in the “molecular_data_cleaning” script of the GitHub repository.

After the datasets are aggregated and preprocessed, Extended-Connectivity Fingerprints (ECFP4) [29] are used to represent each molecule as a binary vector of 1024 length. The fingerprints were extracted using RDKit and DeepChem version 2.5.0. The scripts for the pre-processing and fingerprint extraction are available on the GitHub repository alongside the data. This binary fingerprint represents existence or non-existence of certain sub-graphs within each molecule, with “one” denoting existence of a sub-structural and “zero” denoting its lack of existence. Given two similar molecules, the extracted fingerprints share most of the same positions for “ones” within the binary vector. To calculate how similar two binary fingerprints are Tanimoto score can be used, which is defined as the number of overlapping “ones” divided by the union of “ones” within both fingerprints. This score is used in this work to find the similarity between all molecules within the MolData dataset.

To find the diversity of all molecules within the dataset, first the ECFP fingerprints are calculated. Then, the Tanimoto Similarity Coefficient is calculated between one molecule and the rest of the dataset. The highest Tanimoto Coefficient is then recorded for that molecule, which demonstrates how close the most similar molecule is to the selected molecule. This process is repeated for all molecules and the cumulative histogram of this score is defined. If a large portion of the molecules result in higher Tanimoto Coefficients (higher than 0.7), the dataset as a whole becomes a collection of similar molecules, which in turn hurts the diversity of the dataset. Due to computational constraint caused by calculating the Tanimoto Coefficient matrix, 200,000 molecules were sampled to represent the dataset and this process was implemented on these sampled molecules. ECFP fingerprint extraction as well as Tanimoto Coefficient calculation are made available in the “fingerprint_extraction” script within the GitHub repository.

#### Correlation analysis

To find correlating bioassay, the bioactivity labels of all molecules are taken as representing vectors of each bioassay. To begin, the shared labels between two bioassays that are non-missing are found. The Pearson correlation coefficient is calculated between these two vectors. This process is repeated for all bioassays within each disease category, as well as all the data. The resulting matrices are depicted in the result section. In order to find interesting correlations, the bioassays with a correlation coefficient higher than 0.5 or lower than -0.5 are selected. If the AID number of these bioassays are within 5 of each other (neighbors), they are dismissed, because in most cases they are very closely related screens. The remaining bioassays are further examined to check for any biological cause for this correlation. Correlation calculation is made available in the “correlation” script within the GitHub repository.

#### Classification and performance benchmark

After the data is categorized based on their related diseases, using DeepChem the data is split into training, validation, and test sets, with 80, 10, 10 percent shares respectively. This splitting is done after finding the Bemis-Murcko scaffold of each molecule y [75], and molecules with shared scaffolds are put into same splits. Splitting based on the scaffolds creates more distinct splits, making the problem of classification harder and more like real-world scenarios where the inference set can often have a different distribution than the training set. Having split the data, some tasks may have no positive data points in the smaller splits, which creates a problem for calculating performance metrics, therefore, those tasks are identified, and one of their positive datapoints from the training set is randomly moved to the smaller split. Data splitting is offered in the “splitting” script within the GitHub repository.

The molecules are featurized and converted into graphs with the chirality included in the features. DeepChem was used to featurize the molecules and convert them into undirected graphs with nodes representing atoms and edges representing bonds. These graphs are computationally represented as two matrices: the connectivity matrix and the feature matrix. The feature matrix includes 75 features for each node (atom) within the graph, which include one-hot encoding of the atom type, number of directly bonded neighbors, number of implicit Hydrogens on the atom, formal charge, number of radical electrons, one-hot encoding of the atom's hybridization, and aromaticity. DeepChem also has the option to add chirality features to the feature vectors, which adds three additional values to each vector (78 features in total) representing if the chirality property exists and if so, the classification of the chirality to right-hand or left-hand. The script for featurization of the molecules is available in the “training” scripts within the GitHub repository.

To assist the process, the training split is balanced using weight transformers that affect how the loss is aggregated, amplifying the effect of positive samples during training. The training split is used to train a GCNN in a multitask manner for each category, including one model trained on 600 bioassays combined. The parameters for training and the related model are shown in Table 5. The script for training the GCNN model is made available in the “training” scripts within the GitHub repository.

The evaluation metrics for the training of the models selected in this work are accuracy, recall, precision, and Area Under the Receiver Operator Curve (ROC AUC). While accuracy is a palpable metric of performance, it is not suitable for comparing models in imbalanced scenarios, where ROC AUC can correctly represent performance. Moreover, recall and precision are important in evaluating virtual screening models, since recall denotes how many of the valuable active molecules were correctly predicted, while precision demonstrates how well the trained model can do at inference time, selecting active molecules from a plethora of possible candidates for screening.

To further assess the values of the benchmark and to train another model on the MolData dataset, a fully connected neural network is also trained on all categories. The input to this model is the ECFP4 features of the molecules (binary vectors of length 1024), similar to how traditional virtual screening model are trained. Having this approach allows us to compare the deep learning-based method of virtual screening via automatic feature extraction, to a traditional virtual screening where feature extraction and classification are isolated. The script for training the ECFP based model is made available in the “training” scripts within the GitHub repository.

## Supplementary Information

**Additional file 1.** MolData Data. The main training dataset consisting of 1.4 million molecules with 600 label columns (all_molecular_data). Moreover, the mapping between the 600 datasets and their corresponding disease (aid_disease_mapping) and target (aid_target_mapping) categories.

**Additional file 2.** Data Summary. Summary of all datasets including their AID number, descriptions, statistics, and their disease and target category.

**Additional file 3.** Additional Reference List. A comprehensive list of references to the PubChem datasets used within MolData as well as their corresponding ChEMBL datasets.

**Additional file 4.** Interesting Correlations. Interesting linear correlations found between the labels of different datasets.

**Additional file 5.** Detailed Results. Detailed results of all models and benchmarks.

**Additional file 6.** Additional Results. Training Results for the simple fully connected neural network.

**Additional file 7.** Tagging Keywords. All keywords used for tagging a dataset with a disease benchmark label (i.e., cancer, nervous system, etc.).

## Data Availability

Moldata is available at Additional file 1 as well as an opensource GitHub repository at: https://GitHub.com/Transilico/MolData. The initial version of this repository has also been archived on Zenodo at: https://doi.org/10.5281/zenodo.6021605.
