# Supplementary material for: MolData, a molecular benchmark for disease and target based machine learning
Source: J Cheminform. 2022 Mar 7;14:10. doi: 10.1186/s13321-022-00590-y (PMC8899453; doi:10.1186/s13321-022-00590-y)
Supplement: Supplementary file 6 — Additional file 6. Additional Results. Training Results for the simple fully connected neural network. [file 13321_2022_590_MOESM6_ESM.docx]

**1 - Investigation of Different Multitask Learning Models**

MolData offers an aggregation of 600 datasets as well disease or target tags for each dataset (task). For example, there are many tasks within the overall dataset that have both a disease-related tag as well as a target-related tag. Tasks can be grouped together in four manners: all tasks (600 tasks), all tasks with targets (383 tasks), each task with its own disease category (e.g., cancer tasks), and each task with its own target category (e.g., membrane receptor tasks). In multitask learning setting, these tasks are used for training multiple models including models trained on each disease category, each target category, and aggregation of all tasks with or without targets. Due to the repetition in training, different models’ performance on these shared tasks can be compared to assess which multitask learning model was able to perform the best on each task. The results from this comparison are shown in Figure 7.


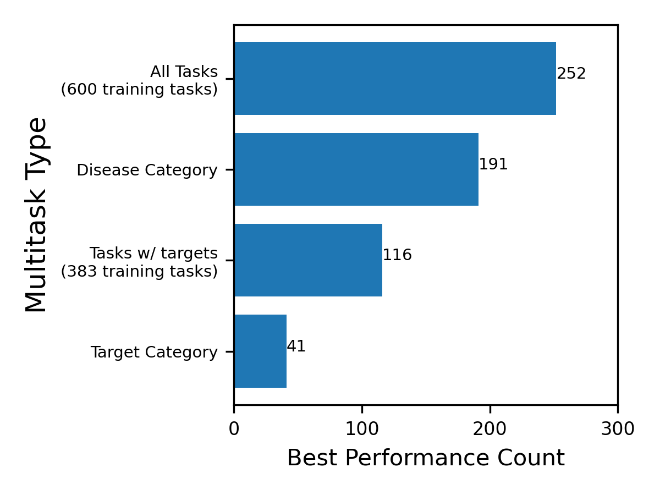


Figure S1 - Comparison of different multitask models for all tasks regarding the number of tasks where each model was the best performing

As shown in Figure S1, 252 tasks have the highest performance when they are combined with all available tasks and used to train a model on all 600 tasks. However, the remaining tasks (348 tasks) have better performance when their model is trained on fewer tasks, such as the models trained on specific disease or target categories, or the model trained on all tasks with target tags. This demonstrates that multitask learning on fewer tasks may be beneficial in some scenarios. While it is intuitive that grouping different dataset that are biologically related would aid their modeling, as it can be seen from Figure S1 it is not necessarily true in the case training a deep learning model. Investigating the number of tasks and their specific grouping that would results in the best performance for a given task would be an interesting research question which is outside of the scope of this work. MolData groups different tasks together so that the benchmark would have a distinct biological reason, and any improvement on the benchmark values can be directly translated to a real-world field of early drug discovery.

**2 - Classification Results on Fully Connected Neural Network**

To further investigate the performance of different models on the MolData benchmark aside from the Graph Convolutional Neural Network, a fully connected neural network was also trained on the data. However, the features that were input to the model were changed to Extended-Connectivity Fingerprints (ECFP4) of the molecules, which are binary vectors of size 1024. This manner of training is synonymous to the traditional virtual screening models which isolate the feature extraction mechanism from the classification algorithm. The hyper-parameters for training this model are shown in Table S1 below.

Table S1 – Parameters of the training the fully connected neural network model.

| Parameter | Value | Parameter | Value |
| --- | --- | --- | --- |
| Dense Layer Size | 1024 | Dropout | 0.1 |
| Featurizer | ECFP | Learning Rate | 0.0001 |
| Epoch Number | 50 | Batch Size | 128 |

The results for the disease and target benchmark are shown in Table S2 and Table S3.

Table S2 - Classification results on the disease categories, averaged on all tasks within each category

|  | **Validation Set** | | | | **Test Set** | | | |
| --- | --- | --- | --- | --- | --- | --- | --- | --- |
| **Disease Benchmark** | **Accuracy (%)** | **Recall (%)** | **Precision (%)** | **ROC AUC** | **Accuracy (%)** | **Recall (%)** | **Precision (%)** | **ROC AUC** |
| **All Tasks** | 84.06 | 54.4 | 4.98 | 0.7698 | 84.24 | 52.74 | 5.23 | 0.7662 |
| **Cancer** | 85.69 | 50.13 | 4.29 | 0.7625 | 85.73 | 48.68 | 4.62 | 0.7571 |
| **Nervous System** | 84.13 | 49.99 | 2.89 | 0.7418 | 84.45 | 48.38 | 3 | 0.7419 |
| **Immune System** | 85.28 | 51.71 | 3.57 | 0.7605 | 85.42 | 47.29 | 3.63 | 0.7491 |
| **Cardiovascular** | 84.24 | 50 | 2.96 | 0.7266 | 84.47 | 47.48 | 3.2 | 0.7332 |
| **Toxicity** | 80.75 | 49.59 | 20.31 | 0.732 | 80.21 | 49.09 | 20.56 | 0.7141 |
| **Obesity** | 86.64 | 51.32 | 5.21 | 0.7679 | 86.65 | 44.47 | 5.09 | 0.751 |
| **Virus** | 86.58 | 45.49 | 3.05 | 0.7425 | 86.64 | 43.58 | 3.21 | 0.7316 |
| **Diabetes** | 84.29 | 53.6 | 5.24 | 0.7663 | 84.43 | 48.26 | 5.36 | 0.747 |
| **Metabolic Disorders** | 84.47 | 47.58 | 6.63 | 0.7387 | 84.71 | 48.04 | 6.6 | 0.7453 |
| **Bacteria** | 89.33 | 49.46 | 5.24 | 0.7713 | 88.99 | 48.39 | 5.32 | 0.7779 |
| **Parasite** | 84.9 | 57.92 | 6.14 | 0.7999 | 85.13 | 55.15 | 6.28 | 0.7998 |
| **Epigenetics-Genetics** | 87.31 | 47.02 | 6.4 | 0.778 | 87.42 | 42.66 | 5.45 | 0.7318 |
| **Pulmonary** | 81.96 | 46.04 | 2.83 | 0.6953 | 82.14 | 43.34 | 3.09 | 0.6898 |
| **Infection** | 85.2 | 51.47 | 7.31 | 0.7476 | 85.2 | 50.77 | 7.31 | 0.7716 |
| **Aging** | 94.34 | 27.11 | 2.13 | 0.6488 | 93.98 | 30.73 | 3.83 | 0.7208 |
| **Fungal** | 89.4 | 43.15 | 3.07 | 0.742 | 90.02 | 35.48 | 2.94 | 0.7176 |

Table S3 - Classification results on the target categories, averaged on all tasks within each category

|  | **Validation Set** | | | | **Test Set** | | | |
| --- | --- | --- | --- | --- | --- | --- | --- | --- |
| **Target Benchmark** | **Accuracy (%)** | **Recall (%)** | **Precision (%)** | **ROC AUC** | **Accuracy (%)** | **Recall (%)** | **Precision (%)** | **ROC AUC** |
| **All Tasks w/ Targets** | 83.87 | 54.77 | 5.33 | 0.768 | 84.03 | 52.95 | 5.5 | 0.7663 |
| **Membrane receptor** | 84.27 | 45.87 | 2.55 | 0.7149 | 84.98 | 43.23 | 2.46 | 0.7111 |
| **Enzyme (other)** | 87.34 | 54.39 | 3.36 | 0.7963 | 87.23 | 52.07 | 3.55 | 0.7794 |
| **Nuclear receptor** | 79.08 | 57.79 | 15.4 | 0.7503 | 78.9 | 52.85 | 14.44 | 0.7374 |
| **Hydrolase** | 88.48 | 53.22 | 3.87 | 0.7976 | 88.42 | 48.11 | 3.87 | 0.7709 |
| **Protease** | 86.65 | 47.32 | 3.47 | 0.7407 | 86.61 | 45.99 | 3.83 | 0.759 |
| **Transcription factor** | 83.03 | 52.87 | 12.36 | 0.7517 | 82.98 | 52.21 | 13.08 | 0.7633 |
| **Kinase** | 85.15 | 42.42 | 2.75 | 0.702 | 85.42 | 37.12 | 2.19 | 0.6688 |
| **Epigenetic regulator** | 85.74 | 50.8 | 4.91 | 0.7601 | 84.9 | 48.82 | 5.2 | 0.7547 |
| **Ion channel** | 86.61 | 42.03 | 2.36 | 0.7117 | 86.86 | 43.2 | 2.59 | 0.7371 |
| **Transferase** | 89.16 | 54.58 | 5.38 | 0.8047 | 88.82 | 51.82 | 4.36 | 0.7854 |
| **Oxidoreductase** | 87 | 49.78 | 8.32 | 0.7662 | 87.16 | 48.38 | 7.37 | 0.7912 |
| **Transporter** | 87.2 | 33.82 | 1.96 | 0.6343 | 87.51 | 32.13 | 1.98 | 0.6176 |
| **NTPase** | 85.25 | 48.93 | 13.03 | 0.6672 | 85.4 | 52.68 | 13.84 | 0.7639 |
| **Phosphatase** | 88.73 | 46.13 | 2.69 | 0.7636 | 88.55 | 45.9 | 2.78 | 0.7391 |

As it can be seen from Table S2 and Table S3, the finger print based fully connected neural network has slightly worse performance compared to the GCNN model, while following the same trend in all cases in regards the absolute values (compared to the GCNN model).
